# Acknowledgement to Reviewers of *Biomedicines* in 2019

**DOI:** 10.3390/biomedicines8010017

**Published:** 2020-01-19

**Authors:** Biomedicines Editorial Office

**Affiliations:** MDPI, St. Alban-Anlage 66, 4052 Basel, Switzerland

The editorial team greatly appreciates the reviewers who have dedicated their considerable time and expertise to the journal’s rigorous editorial process over the past 12 months, regardless of whether the papers are finally published or not. In 2019, a total of 95 papers were published in the journal, with a median time to first decision of 19.59 days and a median time from submission to publication of 36 days. The editors would like to express their sincere gratitude to the following reviewers for their generous contribution in 2019:
Agrahari, VivekManfredini, StefanoAhmed, ZubairMangaonkar, AbhishekAi, YongMarino Gammazza, AntonellaAl-Hilaly, YoussraMartinez, VicenteAmaradhi, RadhikaMartínez-Galiano, Juan MiguelAmato, FeliceMazur-Bialy, Agnieszka IrenaAmezcua, LilyanaMcCaughan, Geoffrey WilliamAmmendola, MicheleMcCombe, PamelaAmpatzis, KonstantinosMei, YangAndjelkovic, Anuska V.Mejias Luqu, RaquelAndreotti, GiuseppinaMerigo, ElisabettaAngrand, Pierre-OlivierMira, José JoaquínAnsari, RaisMiron, Richard J.Arciello, AngelaMizuo, KeisukeAshaie, MaeirahMonguió-Tortajada, MartaAvin, VijayMunteanu, Florentina-DanielaBak, IstvanMurray, Peter E.Balan, SreeKumarMusgrave, IanBalcerczyk, AnetaNeuman, ManuelaBaldwin, Albert S.Niikura, RyotaBanchelli, MartinaNitulescu, MihaiBanerjee, AditiNorihiko, NaritaBarba, ClaraNouri, AriaBeharry, Kay D.Nowak, AdrianaBehrens, JürgenNyati, Kishan KumarBeis, DimitrisOchieng, JosiahBhandari, Manohar PrasadOdland, JonBiagini, GiuseppeOlszak, TomaszBotelho, MonicaOmar, Syed HarisBranca, Jacopo Junio ValerioOng, Albert C. M.Cabaro, SerenaOsuagwu, BethelCakstina, InesePan, YangangCapasso, RaffaelePapantonopoulos, GeorgeCattò, CristinaPark, Jun-BeomChahin, SalimPatrucco, LilianaChalah, Moussa AntoinePatti, FrancescoChang, Jer-MingPessina, FedericoChang, Tsung-HsienPincus, SethChilaka, Cynthia AdakuPorebski, GrzegorzChirumbolo, SalvatorePorzionato, AndreaChoi, Eung HoQie, YaqingChoukroun, JosephRanzato, EliaConlon, MichaelRaposo, MariaDandawate, PrasadRavula, AbhigyanDangol, ManitaRiffelmacher, ThomasDas, AmitavaRivera, Víctor M.Di Maro, AntimoRočka, SauliusEar, JasonRomero, Marta R.Exbrayat, Jean-MarieRose, PeterFailla, Cristina M.Royo, FelixFitzgerald, DavidRudolf, RebekaForn, CristinaRuiz Téllez, TrinidadFrank, SašaSahu, Saura C.Freedman, MarkSakamuri, Siva Sankara Vara PrasadFujihara, HisakoSalim, VonnyFuller, BryanSantacroce, LuigiGajęcki, MaciejScarpino, StefaniaGantenbein, BenjaminSchaiquevich, PaulaGaranto, AlejandroSeedorf, UdoGarcía, TaniaSeitz, Helmut K.Garin, Marina I.Shahi, ShaileshGarofalo, MariangelaSharma, GayatriGarrity, DeborahSharma, ShwetaGea Sanchez, AlfredoSimon, Marie ChristineGentilucci, LucaSinha Ray, ShresthaGHILA, LuizaSips, PatrickGilli, FrancescaSkowron, KrzysztofGohda, EiichiSong, Yang-HeonGolubovskaya, VitaSorg, OlivierGris, DenisSplichal, IgorGrubić Kezele, TanjaSrivastava, PranayGupta, RajatStange, KatjaGupta, VijayalaxmiStepanenko, Aleksei A.Hellinger, RolandStratmann, BerndHelm, RichardStruzik, JustynaHilhorst, MarcSullivan, ConHossain, Md KamalSung, Myong-HeeHuang, Chih-LingTadesse, MelkieIppoliti, RodolfoTakano, KatsuraJayaprakash, PriyamvadaTanner, Julian A.Jazvinšćak Jembrek, MajaTartey, SarangJaźwińska, AnnaTomita, HiroyukiJohnson, LorraineTsushima, TakahiroJung, YoungmiTzeng, Shiang-JongKanamori, TakaoVan Der Velde, EnnoKaur, GurvinderVarricchio, EttoreKeightley, CristinaVeltkamp, ChristianKoh, Cho YeowVerma, SmritiKoide, TetsuyaVersino, ElisabettaKoller, GabrieleVishe, MaheshKozłowska, MariolaVymetalkova, VeronikaKuryk, LukaszWallace, David R.Kushkevych, IvanWaxman, JoshuaKutikhin, Anton G.Wilhelms, Daniel B.Laakkonen, Johanna P.Wilkinson, AnneLa-Beck, NinhWodrich, HaraldLam, Yuen TingWood, AlasdairLeporatti, StefanoXin, GangLevitsky, Victor G.Yagmur, ErayLi, GuohuiYe, ZhennanLoosli, FelixYip, Bon HamLuna, AurelioYuan, ZhihongMa, WanshuYuann, Jeu-Ming P.Madine, JillianYura, YoshiakiMajumder, PoulamiZaprutko, LucjuszMandato, ClaudiaZhang, Guanshi

